# Registered Replication Report of Weissman, D. H., Jiang, J., & Egner, T. (2014). Determinants of congruency sequence effects without learning and memory confounds

**DOI:** 10.3758/s13414-020-02021-2

**Published:** 2020-09-15

**Authors:** Mate Gyurkovics, Marton Kovacs, Matt Jaquiery, Bence Palfi, Filip Dechterenko, Balazs Aczel

**Affiliations:** 1 Beckman Institute, University of Illinois at Urbana-Champaign, 405 N Mathews Ave, Urbana, IL 61801 USA; 2 Institute of Psychology, ELTE, Eotvos Lorand University, Budapest, Hungary; 3 Department of Experimental Psychology, University of Oxford, Oxford, UK; 4 School of Psychology, University of Sussex, Brighton, UK; 5 Sackler Centre for Consciousness Science, University of Sussex, Brighton, UK; 6 Institute of Psychology, Czech Academy of Sciences, Prague, Czech Republic

**Keywords:** Registered replication, Congruency sequence effect, Cognitive control, Online data collection

## Abstract

The congruency sequence effect (CSE) refers to the finding that the effect of cognitive conflict is smaller following conflicting, incongruent trials than after non-conflicting, congruent trials in conflict tasks, such as the Stroop, Simon, and flanker tasks. This is typically interpreted as an upregulation of cognitive control in response to conflict. Weissman, Jiang, & Egner (2014) investigated whether the CSE appears in these three tasks and a further variant where task-irrelevant distractors precede the target (prime-probe task), in the absence of learning and memory confounds in samples collected online. They found significant CSEs only in the prime-probe and Simon tasks, suggesting that the effect is more robust in tasks where the distractor can be translated into a response faster than the target. In this Registered Replication Report we collected data online from samples approx. 2.5 times larger than in the original study for each of the four tasks to investigate whether the task-related differences in the magnitude of the CSE are replicable (Nmin = 115, Nmax = 130). Our findings extend but do not contradict the original results: Bayesian analyses suggested that the CSE was present in all four tasks in RT but only in the Simon task in accuracy. The size of the effect did not differ between tasks, and the size of the congruency effect was not correlated with the size of the CSE across participants. These findings suggest it might be premature to conclude that the difference in the speed of distractor- vs target-related response activation is a determinant of the size of cross-trial modulations of control. The practical implications of our results for online data collection in cognitive control research are also discussed.

**Electronic supplementary material:**

The online version of this article (10.3758/s13414-020-02021-2) contains supplementary material, which is available to authorized users.

Cognitive control is an umbrella term for multiple processes that all play a role in setting up and changing attentional weights in order to generate a goal-relevant response (Gratton, Cooper, Fabiani, Carter, & Karayanidis, 2018). One component process is the inhibition of task-irrelevant responses, which is often assessed with conflict paradigms (Gratton et al., 2018). In these tasks, participants are required to respond to a task-relevant stimulus (or stimulus feature) in the presence of task-irrelevant stimuli (or stimulus features). On congruent trials, distracter stimuli cue the same response as the target stimulus (e.g., HHH, where the task is to identify the central target letter), while on incongruent trials the distracter stimuli prime an incorrect response (e.g., GHG). Participants tend to perform worse – more slowly and less accurately – on incongruent trials, compared to congruent trials, a phenomenon known as the congruency or interference effect.

Importantly, in a seminal paper Gratton, Coles, & Donchin (1992) demonstrated that the size of the congruency effect on trial N is also modulated by the congruency of trial N-1. Specifically, the congruency effect is smaller following incongruent trials compared to congruent trials (congruency sequence effect, CSE). Since its initial demonstration, numerous theories have been put forward to account for this finding (for a review see e.g., Duthoo, Abrahamse, Braem, Boehler, & Notebaert, 2014a), the most prominent of them being the conflict monitoring hypothesis (Botvinick, Braver, Barch, Carter, & Cohen, 2001). This posits that when conflict is encountered in the processing stream, cognitive control is upregulated leading to lower interference on the subsequent trial. In other words, this account explains the CSE in terms of a conflict-triggered top-down adjustment of cognitive control.

This top-down interpretation of the effect, however, is often complicated by the presence of confounds in the task design (Duthoo et al., 2014a). For instance, in conflict tasks such as the Stroop (Stroop, 1935), flanker (Eriksen & Eriksen, 1974), or Simon task (Simon & Small, 1969), there are four types of trial transitions from trial N-1 to trial N based on the combination of incongruent and congruent trials. Transitions can also be categorized in terms of feature repetition: complete alternations (no overlap in features from trial N-1 to trial N; e.g., GGG→HHH), complete repetitions (two identical trials in a row; e.g., GGG→GGG), and partial repetitions (the two trials share one feature; e.g., GHG→HHH). The difficulty in interpretation stems from the fact that the two classifications of transitions are not independent; for example, in *two-choice* variants of these tasks, complete repetitions can only occur when congruency repeats, while all congruency switch transitions will be partial repetitions (Mayr, Awh, & Laurey, 2003). Due to episodic memory-based feature integration effects (detailed in Hommel, Proctor, & Vu, 2004), this confound could account for the CSE pattern without the necessity of invoking top-down control mechanisms at all.

A simple solution to the feature integration confound is to increase the stimulus set and the response set, for example from 2 to 4, and prevent repetitions, or exclude them from the analyses. This, however, creates a different problem (Mordkoff, 2012; Schmidt & De Houwer, 2011). For example, if the proportion of congruent trials is to be kept at 50% in a four-choice task, the frequency of each congruent stimulus combination has to be inflated compared to what would be expected if stimulus features were combined randomly; for example, HHH would appear three times as often as either KHK, GHG, or DHD. Consequently, the distracter H would be associated with the response H more frequently than with any other response. In other words, a contingency would exist between the irrelevant dimension and the correct response, making the former informative, and not truly task-irrelevant. Schmidt, Crump, Cheesman, & Besner (2007) demonstrated that not only do individuals react faster and more accurately to high-contingency (highly predictive) trials than to low-contingency trials, the size of this contingency effect is also modulated by previous trial contingency. As in 50% congruent four-choice conflict tasks congruency is perfectly confounded with contingency, this contingency sequence effect could also be partly – or entirely – responsible for the typical CSE pattern.

Early studies controlling for these confounds either post-hoc (i.e., by removing trials where features have been repeated, e.g., Mayr et al., 2003; Nieuwenhuis et al., 2006) or by preventing them from occurring by design (e.g., Mayr et al., 2003) failed to detect the CSE effect in traditional Stroop and flanker tasks. However, the effect was found using prime-probe tasks, where the presentation of the distracter – or distracters - precedes the presentation of the target (Kunde & Wuhr, 2006; Schmidt & Weissman, 2014).

## Weissman, Jiang, & Egner’s (2014) Original Study

Weissman et al. (2014) conducted a highly extensive online study on the Amazon Mechanical Turk crowdsourcing platform to investigate the question why the CSE is more likely to appear in prime-probe tasks than in classical conflict tasks in the absence of learning and memory confounds. An additional goal was to validate the use of online data collection in cognitive control research.

Three experiments were carried out to explore the theoretical research question. In Experiment 1, participants completed a prime-probe task. In Experiment 2, three groups of participants completed confound-free versions of the Stroop, flanker, or Simon task in a between-subject design. Finally, in Experiment 3 a prime-probe variant of the flanker task (temporal flanker task) was used, with the additional between-subject manipulation of whether the line of distracters that appeared ahead of the target contained a central distracter (i.e., a distracter in the location of the upcoming target) or not.

The CSE pattern was found in the prime-probe and Simon tasks, and the temporal flanker task but only if the distracters overlapped with the upcoming target spatially. No CSE was detected in the Stroop and flanker tasks. The authors concluded that two preconditions need to be met for the CSE to appear in a task: 1) stimulus-to-response (S-R) translation can be completed more quickly for the distracter than for the target, and 2) there is spatial overlap between the distracter and the target.

Weissman et al. (2014) interpreted these constraints in terms of the activation-suppression hypothesis. This account posits that presenting the distracter before the target leaves more time for the inhibition of the distracter-related response. This suppression is further enhanced on trials following incongruent compared to congruent trials due to the recent – previous-trial - inhibition of the pathway through which distracter-related responses are activated (Burle, van den Wildenberg, & Ridderinkhof, 2005). Thus, the activation suppression framework can successfully account for the pattern of the CSE, and can also provide a plausible explanation for why the prime-probe and Simon tasks are more likely to engender it. Although this interpretation is consistent with a top-down control-based account of the CSE, it differs from the conflict monitoring account in how control is implemented.

Later laboratory studies, however, found the CSE in flanker and Stroop tasks as well, using confound-free designs similar to those used by Weissman et al. (2014) online (e.g., flanker: Kim & Cho, 2014; Weissman, Colter, Drake, & Morgan, 2015; Stroop: Duthoo, Abrahamse, Braem, Boehler, & Notebaert, 2014b; Stroop and flanker: Aschenbrenner & Balota, 2017). Therefore, it seems likely that temporal difference in distracter- and target-related S-R translation (the distracter “head-start”) is not a necessary precondition of the CSE but simply a determinant of the effect’s magnitude (Weissman, Egner, Hawks, & Link, 2015).

## The Present Study

In the present registered replication report, we aimed to replicate the findings of Experiments 1 and 2 of the original study by Weissman et al. (2014) using larger samples, for two key reasons.

First, our results could provide support for the notion that the size of the CSE differs systematically across tasks. This could catalyse further research into the determinants of the size of the effect. If the distracter head start hypothesis is correct, we would expect the CSE to be larger in the prime-probe and Simon tasks compared to the Stroop and the flanker, in accordance with Weissman et al.’s (2014) original findings. We will also investigate whether the magnitude of interference predicts the magnitude of the CSE. Weissman et al. (2014) found no consistent relationship between these two variables across tasks, suggesting that conflict magnitude is not a strong predictor of the size of the CSE in contrast with the predictions of the conflict monitoring hypothesis (Botvinick et al., 2001). Importantly, however, we will use Bayesian statistics alongside more traditional frequentist statistics in these – and all other - analyses, which will allow us to determine if the absence of a significant relationship is truly evidence of the absence of an effect (Dienes, 2014).

Second, our replication will have important practical implications too. It will help determine the effect size of the CSE in online versions of four different confound-free tasks frequently used in cognitive control research. This is important as online data collection is cheaper and more efficient than in-lab data collection, and it allows access to larger populations (Reips, 2000). Task-specific effect sizes are crucial pieces of information for researchers who are planning to conduct cognitive control research online, as they can help optimize the design of studies. For instance, it is possible that a CSE can be observed online in the flanker and Stroop tasks as well – just as it can be in laboratory tasks – but the effect is of such a small magnitude that sample sizes would have to be unreasonably high to detect it in this somewhat less-controlled setting.

## Methods

### Participants

The original study aimed to collect 50 participants per task in Experiments 1 and 2, resulting in an N of 43 in Exp. 1 (one task only), and a total N of 130 in Exp. 2 (43 for the Stroop, 41 for the flanker, and 46 for the Simon). In our replication, target N per task was 2.5 times that of the original target (2.5 × 50 = 125), following the guidelines suggested by Simonsohn (2015). Data collection was stopped once target Ns were reached.

Participants were recruited online, by two collaborating laboratories from Hungary and the Czech Republic. Each participant received compensation, such as course credit for taking part. Table 1 summarizes the final composition of the samples collected for each task.

**Table 1:** Sample Characteristics for the Four Tasks

	Prime-Probe	Flanker	Stroop	Simon
N	115	125	130	119
Mean age (*SD*)	22 (4)	22 (3)	21 (2)	23 (5)
Females (%)	74.11	76.66	80.95	75.86
Hungarian (%)	75.65	81.60	81.54	82.35

Note: Participants were either Czech or Hungarian citizens.

The study was approved by the Departmental Ethics Committee of each collaborating research group.

### Tasks

The original authors provided the JavaScript-based codes they used for data collection. Based on these scripts, new experimental scripts were written for the tasks which can be found at https://github.com/mjaquiery/Weissman-replication. The original instructions were translated from English to Hungarian and Czech.^1^

#### Stimuli

Task parameters of the four tasks including the characteristics of the stimulus and response sets are summarized in Table 2.

**Table 2:** Task Parameters of the Four Tasks used in the Experiment

	Prime-Probe	Stroop	Flanker	Simon
Stimuli	Words: Up, Down, Left, Right	Words: RED, BLUE, GREEN, YELLOW	Letters: M, T, H, S	Coloured squares
Stimulus size	Distracter: 48pt Target: 77pt	72pt	60pt	100 × 100 pixels
Stimulus colours	black	red, blue, green, yellow	black	red, blue, green, yellow
Response keys	F, G, J, N	Z, X, N, M	Z, X, N, M	Left, right, up, down arrows
Number of trials	4 blocks of 97 trials	4 blocks of 81 trials	4 blocks of 81 trials	4 blocks of 81 trials

Note: All stimuli were presented on a grey background. Participants were instructed to use the following fingers for the response keys listed: left middle, left index, right middle, and right index finger, respectively. Each task started with a 24-trial long practice block. In the Stroop, Simon, and flanker tasks, feedback on performance was given after every trial during the practice session, but not during the task sessions. In the prime-probe task, error feedback was provided during the task blocks as well. In the prime-probe and the Stroop tasks, Hungarian translations of the target words were used in the Hungarian subsample: UP = FEL, DOWN = LE, LEFT = BAL, RIGHT = JOBB, and RED = PIROS, BLUE = KÉK, GREEN = ZÖLD, YELLOW = SÁRGA, and the Czech translations of the target words were used in the Czech subsample: UP = NAHORU, DOWN = DOLŮ, LEFT = VLEVO, RIGHT = VPRAVO, and RED = ČERVENÁ, BLUE = MODRÁ, GREEN = ZELENÁ, YELLOW = ŽLUTÁ.

#### Design

**Figure 1:**
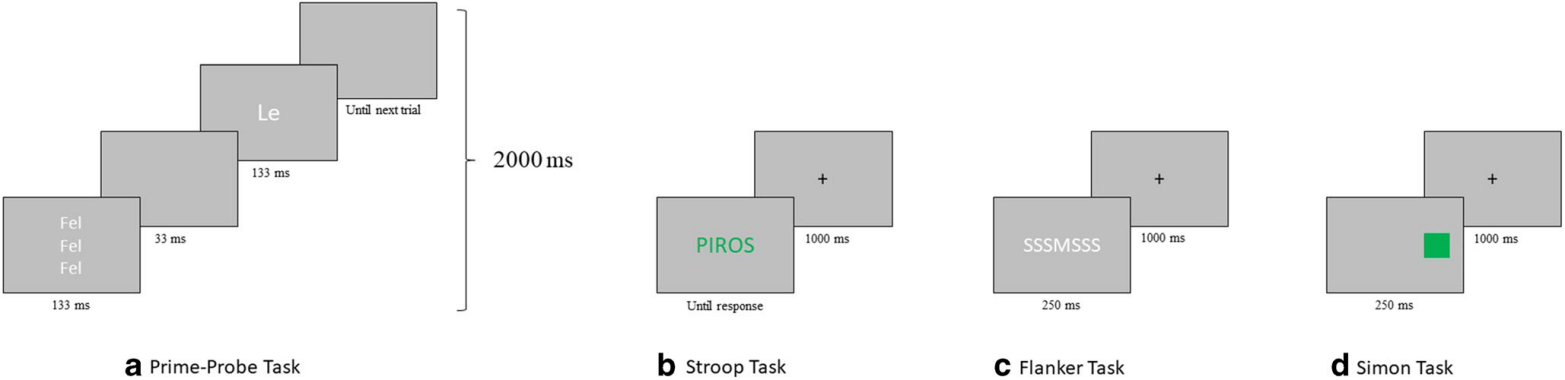
– Examples of trials from each of the four tasks used in the study. (a) The prime-probe task. Stimulus durations were set to 33 ms and 133 ms to correspond to two and eight refreshes, respectively, of a typical, 60 Hz monitor. Fel = Up, Le = Down. (b) The Stroop task. PIROS = RED. (c) The flanker task. (d) The Simon task.

Figure 1 illustrates the events that occurred on a single trial of each task. In the prime-probe task, participants were instructed to identify the single target word presented after the distracters. Distracter stimuli consisted of three words, stacked vertically. The three words were always identical (e.g., the word Up displayed three times). The target word could either be the same word (congruent trials) or a different word (incongruent trials).

In the Stroop task, their task was to identify the colour of the target words, irrespective of the meaning of the word. On congruent trials, the meaning and the colour of the word coincided (e.g., RED in red), whereas on incongruent trials, the two mismatched (e.g., RED in green).

In the flanker task, they had to identify the central letter in a string of 7 letters. The flanking letters were always identical, and their identity could either match that of the central letter (congruent trials; e.g., HHHHHHH) or mismatch (incongruent trials; e.g., HHHMHHH).

In the Simon task, they had to indicate the colour of a square, while ignoring its location. The location of the square could either match the location of the response button on the keyboard associated with the square’s colour (congruent trials), or mismatch (incongruent trials).

In all three tasks feature integration effects and contingency learning were prevented using the same strategy, which we will demonstrate through the example of the prime-probe task. The four stimuli (Up, Down, Left, Right) were divided into two sets (Up/Down and Left/Right), and the trial sequence alternated between these two sets on odd and even trials, so for example, on odd trials, only distracter-target combinations that contained Up and Down could appear, while on even trials, only combinations of Left and Right were used. This way, no features could be repeated from one trial to the next (as adjacent trials used non-overlapping stimulus sets), and the irrelevant dimension was not rendered informative because some unique stimulus combinations were never used (e.g., Up/Left, Up/Right, etc). For both stimulus sets, proportion of congruent trials was kept at 50%.

### Procedure

In accordance with the original study, each participant completed only one of the four tasks. Participants completed the task online, using their own computer, and in their own time. They were sent a brief description of the study, and a link to the task they were randomly assigned to by email. After clicking the link, they completed a consent form, and a brief demographic questionnaire, followed by the instructions to the task. The instructions emphasized that they should not complete the task on a tablet or a smartphone. In all other respects they were identical to the original instructions used by Weissman et al. (2014). Following the instruction and a brief, 24-trial practice session, each participant completed one of the four tasks.

## Analysis plan

### Data pre-processing

We followed the same data pre-processing steps as the original authors. Only task block trials were analysed. All analyses were conducted in R version 3.6.1 (R Core Team, 2019). The tidyverse R packages were used for data pre-processing and data management (Wickham et al., 2019).

Before starting the analyses, we excluded participants whose mean accuracy on their respective task was below 70% and/or whose mean response time (RT) was more than 2.5 *SD*s away from the mean of their group.

As a first step of response time analyses, error trials; trials immediately following errors; outliers, defined as trials 2.5 *SD*s away from the conditional mean of the participant; and trials immediately following outliers were removed. For error analyses, incorrect and post-error trials were not discarded.^2^

Although p-values are also reported, Bayes factors (B) were used to make inferences about the data. As suggested by Jeffreys (1961), a B higher than 3 was taken to indicate good enough support for the alternative model and thus, by symmetry, B lower than 1/3 was considered as good enough evidence for the null model. We used the R code developed by Dienes & Mclatchie (2018) to calculate Bs. Note that the value of the B is subject to the features of the distribution we choose to model the predictions of the alternative hypothesis (Rouder, Morey, Verhagen, Province & Wagenmakers, 2016; Rouder, Morey & Wagenmakers, 2016). Based on the assumption that small effect sizes are more likely to occur than large effect sizes, we employed half normal distributions to represent the predictions of the alternative models (Dienes, 2014). Nonetheless, the *SD* of these distributions can be motivated in multiple ways. Therefore, we report Bayesian Robustness Regions (RR) notated as RR[*SD*_smallest_, *SD*_largest_] including the smallest and largest *SD*s that would bring us to the same conclusion as the B calculated with the chosen *SD*. For instance, for a B larger than 3 the RR would highlight the smallest and largest *SD*s with which the B would be equal to or slightly greater than 3.

### Confirmatory analyses

First, we investigated whether the CSE was present in the different tasks. Two 2 × 2 repeated-measures ANOVAs were run per task, one with mean RT and one with mean accuracy as dependent variable. In both cases, the two factors of the ANOVA were Previous Trial Congruency (congruent, incongruent) and Current Trial Congruency (congruent, incongruent). In these analyses, a half-normal distribution was used with a mode of 0 and a *SD* equal to the half of the congruency effect (in ms) for the particular task in the original data set by Weissman et al. (2014) to model the prediction of the interaction effect. To test the congruency main effect, the *SD* of the H1 model was set to the congruency effect reported by Weissman et al. (2014) for the given task. Significant interactions were followed up by simple effects analyses, contrasting post-congruent congruent (cC) and post-incongruent congruent (iC) trials in one analysis, and post-congruent incongruent (cI) and post-incongruent incongruent (iI) trials in another. The prior H1 model for both was a half-normal distribution with a mode of 0 and an *SD* equal to half of the CSE estimate for that given task, i.e., the originally reported congruency effect divided by four.

Next, we examined whether the size of the CSE varies across tasks. To investigate this, a CSE index was calculated based on each participant’s RT data, using the following formula: (cI – cC) – (iI – iC), where each variable represents the within-subject mean of that particular condition.

A one-way between-subject ANOVA with task (levels: Prime-Probe, Flanker, Stroop, Simon) as the single factor, and the CSE index as the dependent variable was then run to examine if the size of the effect differs across tasks. If the Levene test indicated the violation of the assumption of homogeneity of variances, a Kruskal-Wallis H test was run instead. In the former case, Tukey’s post-hoc test was used for pairwise comparisons, while in the latter, Dunn’s test was run. For pairwise comparisons, a half-normal distribution was used with a mode of 0 and *SD* equal to half of the greater congruency effect of the two in any given pair as reported by Weissman et al. (2014).

Finally, to investigate whether the size of the CSE changes as a function of the magnitude of interference, correlations between the CSE index and the congruency effect – calculated by subtracting the participant’s mean congruent RT from their mean incongruent RT - were calculated, within each task. Bs were calculated based on the Fisher *Z* transformed *r*-values, and a two-tailed normal distribution was used as a prior, with a mode of 0 and a *SD* of 0.549 (corresponding to an *r* of 0.5).

Task and analysis scripts are available on the project’s Open Science Framework page (https://osf.io/z27sn/).

## Results

10.16%, 2.34%, 3.70%, and 6.30% of participants were excluded due to accuracy below 70% and/or a mean RT more than 2.5*SD*s from the group mean for the prime-probe, flanker, Stroop, and Simon task, respectively. If a participant accidentally completed multiple tasks or a single task multiple times, only their first response in their assigned task was retained.

On average, 12.97%, 14.77%, 12.36%, and 14.59% of trials were removed from the prime-probe, flanker, Stroop, and Simon task, respectively, because they were outliers (they were more than 2.5*SD*s from the corresponding conditional mean of the participant) or were immediately preceded by an outlier. For RT analyses, error and post-error trials were also removed.

**Figure 2:**
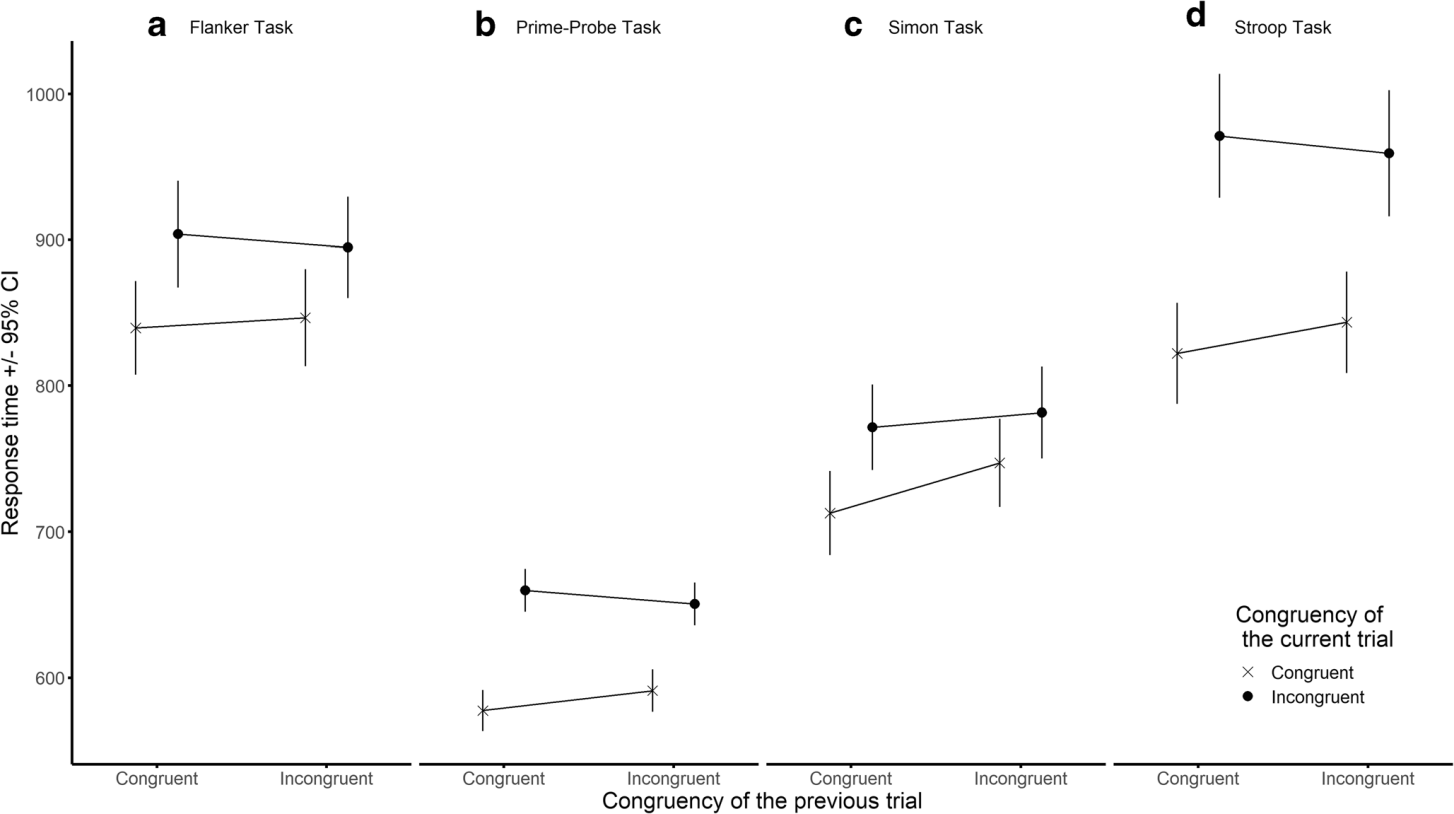
- The congruency sequence effect (CSE) in RT (ms) across the four tasks. Error bars represent 95% confidence intervals.

The results of the ANOVAs investigating RTs of the different tasks are summarized in Table 3. A significant main effect of Current Trial Congruency was found in all four tasks: participants were slower on incongruent compared to congruent trials. The Current Trial Congruency × Previous Trial Congruency interaction in RT (i.e., the CSE) also reached significance in all four tasks. Bayesian analyses of the interaction effect suggested evidence in favour of H1 in every task. Figure 2 shows the pattern of the Current Trial Congruency × Previous Trial Congruency interaction in RT across the four tasks.

**Table 3:** Findings of the Analyses Investigating the Congruency Sequence Effect (CSE) in the Prime-Probe, Flanker, Stroop, and Simon Tasks.

		RT						Accuracy					
		F	*p*	η^2^_p_	B	*SD*(B)	RR	F	*p*	η^2^_p_	B	*SD*(B)	RR
Prime-Probe	CC	689.6	<.001	.96	2.70*10^47^	73.23	[0.76, 2.75*10^4^]	42.36	<.001	.33	1.19*10^7^	0.02	[4.3*10^-4^, 4.32]
	PC	2.80	.097	.03	–		–	5.96	.016	.05	–		–
	CC × PC (CSE)	75.69	<.001	.40	6.12*10^11^	36.62	[0.54, 8468]	2.32	.130	.02	1.62	0.01	[0, 0.058]
Flanker	CC	120.1	<.001	.64	2.66*10^17^	53.62	[0.99, 2.13*10^4^]	3.19	.077	.03	1.83	0.01	[0, 4*10^-3^]
	PC	0.08	.774	<.01	–		–	1.09	.298	.01	–		–
	CC × PC (CSE)	4.55	.035	.04	4.27	26.81	[5.3, 42]	0.18	.673	<.01	0.80	4.8*10^-3^	[0, 0.015]
Stroop	CC	206.9	<.001	.86	7.47*10^25^	92.52	[1.75, 5.08*10^4^]	35.59	<.001	.33	1.39*10^6^	0.01	[5*10^-4^, 4.465]
	PC	1.00	.319	.01	–		–	10.92	<.001	.08	–		–
	CC × PC (CSE)	12.66	<.001	.09	135.40	46.26	[3.3, 2650]	2.65	.106	.02	2.53	4.7*10^-3^	[0, 0.069]
Simon	CC	144.2	<.001	.57	4.36*10^19^	45.24	[0.76, 1.77*10^4^]	50.32	<.001	.58	2.58*10^8^	0.03	[8.4*10^-4^, 9.96]
	PC	39.01	<.001	.23	–		–	0.04	.847	<.01	–		–
	CC × PC (CSE)	10.64	.001	.08	63.51	22.62	[2.9, 840]	7.50	.007	.06	16.80	0.01	[2*10^-3^, 0.11]

Note: CC = Current Trial Congruency, PC = Previous Trial Congruency, B = Bayes factor, *SD*(B) = the *SD* of the half-normal distribution used as the prior to calculate B, RR = the lower and upper boundaries of the robustness regions associated with each B (see text for further explanation), inf = infinity, - = no a priori hypotheses were formed about these cells. Degrees of freedom were (1,114), (1,124), (1,129), (1,118) for the prime-probe, flanker, Stroop, and Simon task, respectively.

Follow-up analyses revealed evidence that RTs were influenced by the congruency of the previous trial on both congruent and incongruent trials in the prime-probe task (*p*s < .001, *B*s > 3.00*10^3^), but only on congruent trials in the Stroop and the Simon tasks (current congruent follow-up: *p*s < .01, *B*s > 75; current incongruent follow-ups: *p*s > .06; findings were inconclusive in the Stroop task, B_H(0, 23.13)_ = 2.07, and supported H_0_ in the Simon task, B_H(0, 11.31)_ = 0.17). Results were inconclusive for both follow-up analyses in the flanker task (*p*s > .09; 1/3 < *B*s < 3).

**Figure 3:**
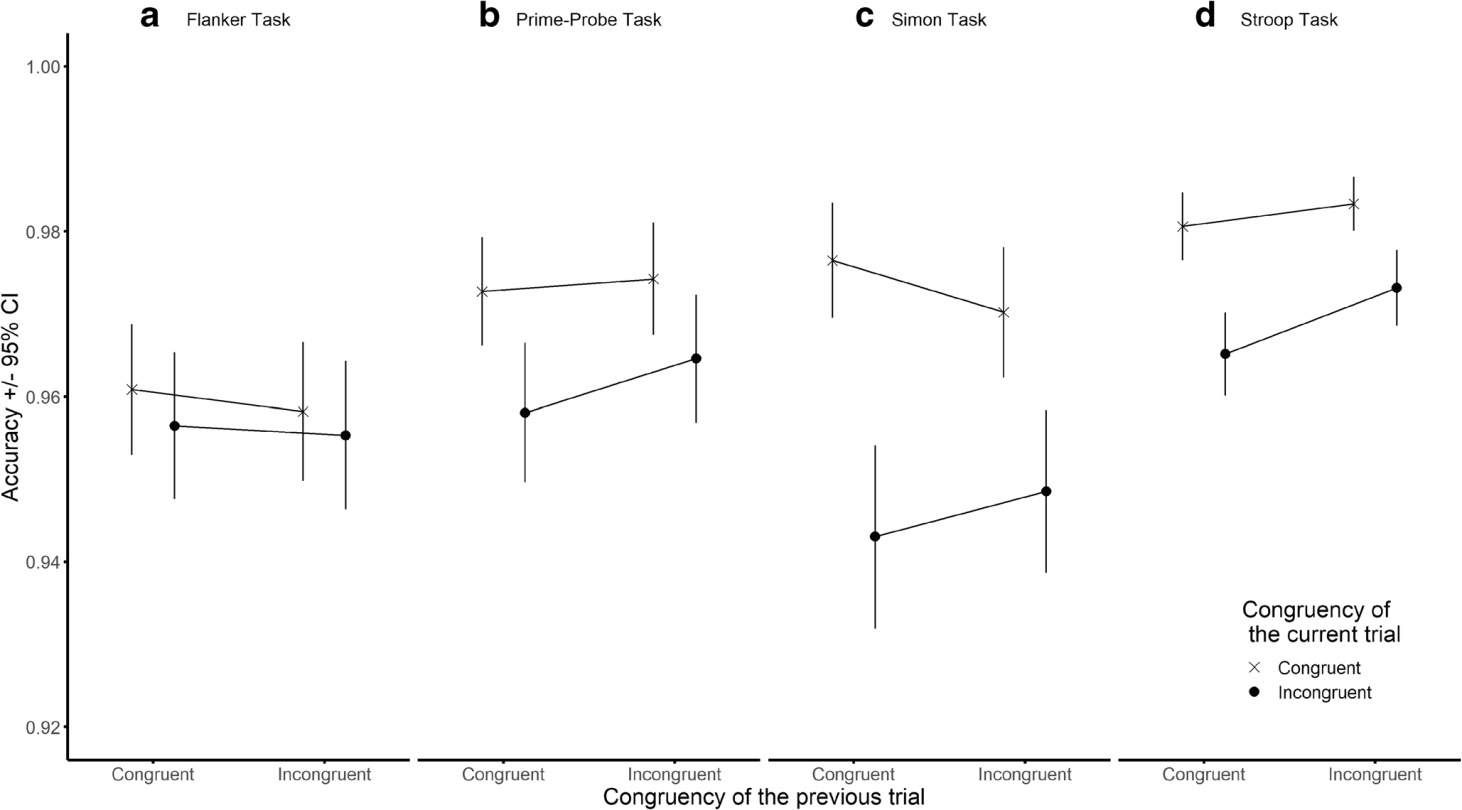
- The congruency sequence effect (CSE) in accuracy (proportion of correct responses) across the four tasks. Error bars represent 95% confidence intervals.

Accuracy as a function of Current Trial and Previous Trial Congruency in the four different tasks is shown in Figure 3. Analyses of accuracy (Table 3) indicated evidence for the main effect of Current Trial Congruency in all tasks except for the flanker task, whereby participants were more error prone on incongruent compared to congruent trials. The CSE was only present in the Simon task in accuracy^3^. Follow-up analyses showed that participants were more accurate on congruent trials following a congruent trial than following an incongruent trial, *t*(118) = 2.59, *p* = 0.011, B_H(0, .007)_ = 12.39, RR[1.25*10^-3^, 0.042].

Next, the magnitude of the CSE in RT across tasks was analysed with a Kruskal-Wallis H test due to unequal variances across groups. The effect of task on the size of the CSE was not significant, *χ*^*2*^(3) = 7.46, *p* = .059. Planned Bayesian pairwise comparisons yielded inconclusive findings for the Stroop vs. prime-probe, Stroop vs. flanker, and flanker vs. Simon comparisons ($$ \raisebox{1ex}{$1$}\!\left/ \!\raisebox{-1ex}{$3$}\right. $$ < *B*s < 3), and support for H_0_ for the Stroop vs. Simon, prime-probe vs. flanker, and prime-probe vs. Simon comparisons (*B*s < $$ \raisebox{1ex}{$1$}\!\left/ \!\raisebox{-1ex}{$3$}\right. $$).

Finally, to investigate the relationship between conflict magnitude and the sequential modulation of control, we examined the correlations between the size of the congruency effect and the size of the CSE in the four tasks. There was a weak positive correlation in the Simon task, *r* = .26, *p* = .004, B_H(0, .549)_ = 16.97 RR[0.042, 3.5]. All other analyses yielded inconclusive findings (*p*s > .05, 1/3 < *B*s < 3) and support for the H_0_ in case of the Stroop task (*B* < 1/3).

Our findings are summarized in Table 4.

**Table 4:** Summary of Our Findings

Task	CSE present for	
	RT	Accuracy
Prime-probe	✓	–
Simon	✓	✓
Stroop	✓	–
Flanker	✓	–

Note: ✓ = support for the presence of the congruency sequence effect (CSE); × = support against the presence of the CSE; – = inconclusive findings.

## Discussion

We aimed to replicate Experiments 1 and 2 from Weissman et al.’s (2014) study that investigated the CSE, a purported index of dynamic adjustments of cognitive control, using confound-minimized variants of four conflict tasks: the prime-probe, the flanker, the Stroop, and the Simon tasks. In the original study, the authors found that a significant CSE emerged in the prime-probe and Simon tasks, but not in the flanker and Stroop tasks in samples collected online. We collected data from substantially (approx. 2.5 times) larger samples using methods that closely followed those of the original study, and our results extend but do not fundamentally contradict the original findings. We found good enough evidence for the CSE in all four tasks in RT, but only in the Simon task in accuracy. The main theoretical focus of our study was to ascertain whether there are stable differences in the size of the CSE across different confound-minimized tasks, however, we were unable to do so as no evidence was found for a task-related effect on CSE magnitude.

Numerically, the CSE in RT was largest in the Stroop task and smallest in the flanker task, with the remaining two tasks falling in between these two in terms of effect size. This order is only in partial agreement with the findings of Weissman et al., who only found significant CSEs in the Simon and prime-probe tasks, but not in the flanker and the Stroop tasks. Consequently, our findings do not provide support for the idea that the CSE is more pronounced in tasks where distractor information can be translated into its corresponding response faster than target information, leading to more efficient inhibition of the distractor pathway. This is because even when considered only numerically, the Simon and the prime-probe tasks do not show larger effects than the Stroop and the flanker tasks. It is, however, worth noting that Gyurkovics, Stafford, & Levita (2020) found the CSE to be smaller in a confound-minimized flanker task compared to a confound-minimized Simon task in a sample of adolescents and young adults. This is in line with the findings of the original Weissman et al. study and the non-significant pattern observed in the present study. While this does suggest that the cross-trial adjustments of control might be smaller in the flanker task than in other classic paradigms, it seems premature to conclude that a difference in speed of processing between distractor and target information is a key determinant of the size of the CSE.

As the effect was present in all four tasks and there was only inconclusive evidence for or even evidence against difference in its magnitude between tasks, our results are not in contradiction with the idea that dynamic adjustments of control are supported by domain-general mechanisms that are engaged similarly in various tasks. However, our study was not designed to address this question directly, and the lack of significant task-related effects in CSE magnitude can be consistent with the existence of multiple conflict-specific control loops that generate similar effects in tasks engendering different types of conflict, but are independent of each other (Egner, 2008). In fact, there is a plethora of empirical findings that support the idea that the mechanism(s) behind the effect may differ across different tasks or may be implemented in a task-specific manner, e.g., findings from studies combining various sources of conflict (Braem, Abrahamse, Duthoo, & Notebaert, 2014; Egner, 2008); investigating age effects in different tasks (Aschenbrenner & Balota, 2017), or the correlation between the size of the effect in different tasks across subjects (Gyurkovics et al., 2020; Whitehead, Brewer, & Blais, 2019). Our results neither support, nor contradict these findings.

In sum, our findings do not suggest that a head start in distracter processing is a prerequisite of the CSE or the determinant of its size as claimed by Weissman et al. (2014). Rather, it appears that the CSE can emerge in all four of the classic conflict tasks investigated by the original authors and our group. What may be the reason for this discrepancy in findings? The most evident answer is statistical power: we used larger samples than Weissman et al., leading to increased power to detect the effect in all four tasks. As such, our results and design provide important information for sample size estimation for future studies aiming to investigate dynamic control adjustments in online samples, using one (or more) of the classic paradigms employed in our study. As mentioned above, the CSE was numerically smallest in the flanker task, a task that has also been found to yield small cross-trial adjustment estimates in previous studies, as such it seems reasonable to advise researchers to use one of the other paradigms – prime-probe, Stroop, or Simon task – in online studies if the main objective of the project is to observe the CSE in the absence of learning and memory confounds, e.g., in studies investigating the magnitude of dynamic control adjustments across different groups.

Our study and Weissman et al.’s original study also differed in the composition of their samples. We conducted our study in the undergraduate student population of two Central European countries (the Czech Republic and Hungary), while the participants of the original study were more diverse in terms of age and ethnicity as they were recruited via Amazon Mechanical Turk, although they were predominantly young adults with a mean age of approximately 30 years. It is possible, albeit speculative, that the undergraduate students in the present study were more highly motivated to perform well on a task presented by a university-related source than Mechanical Turk workers. Future studies could explore the role of achievement motivation on the CSE in various tasks.

It is also worth noting that while there was evidence for the CSE in RT in all tasks, evidence for the effect in accuracy was only present in the Simon task. It is possible that the Simon task was the most difficult for participants, leading to more within-subject variability in performance in terms of accuracy. However, the prime-probe task contained a response deadline element no other task did, consequently larger variability in accuracy could have been expected there as well, yet the CSE was not observed in accuracy in this task. Importantly, as in the original study, the prime-probe task also contained error feedback which might have pushed participants to trade-off speed for accuracy, diminishing variability in the latter variable.

Finally, similarly to the original study, the size of the congruency effect was not consistently correlated with the magnitude of the CSE across tasks in the present study (see also Weissman, Hawks, & Egner, 2016). However, Bayesian analyses suggested that our findings provide no strong evidence either for or against the presence of a relationship in most tasks (with the exception of the Simon task), meaning that such associations might still emerge in even larger samples. Nonetheless, even if future studies were to find evidence for conflict-CSE correlations, the effect sizes of these correlations are likely to be fairly small as even the strongest correlation was only *r* = .26 in our sample. As such, it appears that the magnitude of control adjustment in response to conflict is not clearly dependent on the magnitude of conflict itself when this relationship is examined across subjects. However, it is still possible that control adjustments scale with the magnitude of the conflict signal within individuals, i.e., within-subject fluctuations in conflict could predict within-subject fluctuations of control adjustments strength. Our study was not designed to investigate this question.

In conclusion, using a more powerful design than Weissman et al. (2014), we were able to detect the CSE in RT in confound-minimized variants of four classic conflict tasks in samples collected online, with no substantial difference in the size of the effect across tasks. This set of findings suggests that after careful consideration of the size of the sample available, researchers have a variety of tasks to choose from when investigating dynamic adjustments of control online, as reflected by the CSE in the absence of learning and memory confounds. On a theoretical level, it appears premature to conclude that the size of the CSE is strongly determined by the temporal relationship between distracter and target information.

## Electronic supplementary material

ESM 1 (DOCX 26.8 kb)

## Footnotes

1 During the translation process, instructions were first translated to Hungarian/Czech, then back to English, and finally the re-translated and original texts were compared. All three steps were completed by different authors.

2 Some minor details of the data pre-processing pipeline were not clearly outlined in the original manuscript. These steps include whether participant-level screening based on RT was done before or after discarding the first trial of each block, and whether the second trial of each block was automatically considered a post-outlier trial (and was therefore discarded) or not. We implemented all alternative pipelines in our analysis code to examine if these arbitrary decisions have an impact on our findings. No substantial differences between pipelines were found. Here, we report the findings of the pipeline where participant-level mean RT was calculated after removing the first trial of each block, and second trials were discarded as well.

3 Following the suggestion of an anonymous reviewer, we re-ran the ANOVAs concerning accuracy after excluding trials following errors to control for any post-error control processes. While the effect in the Simon task was noticeably weaker, it was still present, *F*(1,118) = 4.54, *p* = 0.035, B_H(0, .01)_= 4.67, RR[3.2*10-3, 0.025]. We thank the reviewer for bringing this potential confound to our attention.
