# Supplementary material for: Registered Replication Report of Weissman, D. H., Jiang, J., & Egner, T. (2014). Determinants of congruency sequence effects without learning and memory confounds
Source: Atten Percept Psychophys. 2020 Sep 15;82(8):3777–87. doi: 10.3758/s13414-020-02021-2 (PMC7593296; doi:10.3758/s13414-020-02021-2)
Supplement: Supplementary file 1 — (DOCX 26.8 kb) [file 13414_2020_2021_MOESM1_ESM.docx]

**Supplemental Material**

Following the suggestions of an anonymous reviewer, we investigated whether changes in the trade-off between response speed and accuracy across conditions can account for the CSE pattern in RT or accuracy in various tasks. To do this, we calculated balanced integration scores (BIS) in each condition in each task. This measure integrates speed and accuracy with equal weight (Liesefeld & Janczyk, 2019, Behaviour Research Methods). Table 1 summarizes the results of the ANOVAs using BIS as the outcome measure, and Figure 1 illustrates the pattern of our findings in each task. Please note that all of these analyses were exploratory in nature.

Supplemental Table 1 Findings of the Analyses Investigating the Congruency Sequence Effect (CSE) in the Prime-Probe, Flanker, Stroop, and Simon Tasks, using Balanced Integration Scores (BIS) as the Outcome.

|  | **Prime-Probe** | | | **Flanker** | | **Stroop** | | **Simon** | |
| --- | --- | --- | --- | --- | --- | --- | --- | --- | --- |
|  | | **F(1,114)** | **p** | **F(1,124)** | **p** | **F(1,129)** | **p** | **F(1,117)** | **p** |
| **CC** | | 290.90 | < .001 | 28.38 | < .001 | 101.60 | < .001 | 87.36 | < .001 |
| **PC** | | 13.32 | < .001 | 1.89 | .172 | 13.42 | < .001 | 3.78 | .054 |
| **PC x CC** | | 5.72 | .018 | 0.55 | .462 | 4.16 | .044 | 10.31 | .002 |

Note: CC = Current Trial Congruency, PC = Previous Trial Congruency.


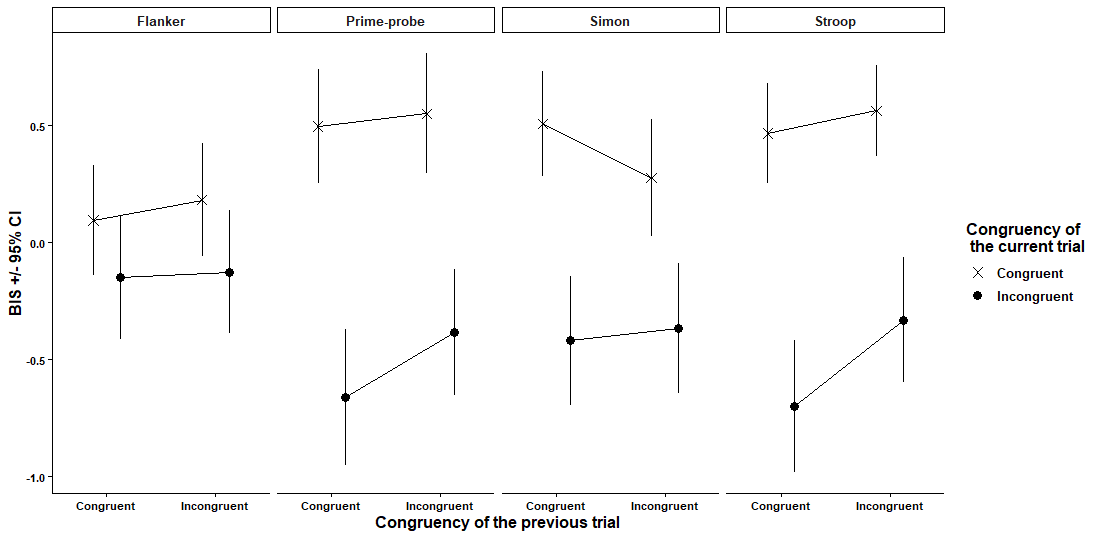


Supplemental Figure 1 – The Congruency Sequence Effect (CSE) in balanced integration scores (BIS) across the four tasks.
